# A Qualitative Study Evaluating the Parent-Specific Cow’s Milk-Related Symptom Score (Pre-CoMiSS™)

**DOI:** 10.3390/nu18142263

**Published:** 2026-07-10

**Authors:** Yvan Vandenplas, Kateřina Bajerová, Christophe Dupont, Mikael Kuitunen, Rosan Meyer, Anna Nowak-Wegrzyn, Carmen Ribes Koninckx, Silvia Salvatore, Raanan Shamir, Annamaria Staiano, Hania Szajewska, Carina Venter, Sue Jones, Catherine Couchepin

**Affiliations:** 1 KidZ Health Castle, UZ Brussel, Vrije Universiteit Brussel, 1090 Brussels, Belgium; 2 Department of Pediatrics, University Hospital Brno, Faculty of Medicine, Masaryk University, 62500 Brno, Czech Republic; bajerova.katerina@fnbrno.cz; 3 Clinique Marcel Sembat, Ramsay Group, 92100 Paris, France; christophe.dupont@wanadoo.fr; 4 Children’s Hospital, Helsinki University Hospital, University of Helsinki, 00014 Helsinki, Finland; mikael.kuitunen@helsinki.fi; 5 Faculty of Medicine, KU Leuven, 3000 Leuven, Belgium; rosan.research@rosan-paediatricdietitian.com; 6 Hassenfeld Children’s Hospital, Department of Pediatrics, NYU Grossman School of Medicine, New York, NY 10016, USA; anna.nowak-wegrzyn@nyulangone.org; 7 Department of Pediatrics, Gastroenterology and Nutrition, Collegium Medicum, University of Warmia and Mazury, 10719 Olsztyn, Poland; 8 Coeliac Disease and Immunopathology Research Unit, La Fe Hospital Research Institute, 46026 Valencia, Spain; carmen_ribes@iislafe.es; 9 Department of Medicine and Technological Innovation, Pediatric Unit, University of Insubria, 21100 Varese, Italy; silvia.salvatore@uninsubria.it; 10 Institute of Gastroenterology, Nutrition and Liver Diseases, Schneider Children’s Medical Center of Israel, Petah Tikva 4920235, Israel; raanan@shamirmd.com; 11 Gray Faculty of Medical and Health Sciences, Tel Aviv University, Tel Aviv 6997801, Israel; 12 Department of Translational Medical Sciences, University of Naples “Federico II”, 80138 Naples, Italy; staiano@unina.it; 13 Department of Paediatrics, Medical University of Warsaw, 02-091 Warsaw, Poland; szajewska@gmail.com; 14 Section of Allergy and Clinical Immunology, Children’s Hospital Colorado, University of Colorado, Aurora, CO 80045, USA; carina.venterrd@gmail.com; 15 Nestlé Nutrition and Health, 1800 Vevey, Switzerland; susan.jones@nestle.com; 16 Ipsos SA, 1219 Geneva, Switzerland; catherine.couchepin@ipsos.com

**Keywords:** cow’s milk, cow’s milk allergy, food allergy, infant, CoMiSS™, digital tool

## Abstract

**Background/Objectives**: Cow’s milk allergy (CMA) is one of the most frequent food allergies in early childhood, yet its diagnosis remains a challenge. The Parent-Specific Cow’s Milk-Related Symptom Score (Pre-CoMiSS™) was developed in 2024 to assist parents in tracking possible cow’s milk-related symptoms in their infants. The aim of this qualitative study was to explore users’ experience with the Pre-CoMiSS™ tool, learn from their experience and identify areas for improvement. **Methods**: Participants were parents of infants aged up to 12 months who felt that their babies were experiencing possible feeding-related symptoms. Participants were selected from a local panel of potential parents in the UK and Sweden. Forty-five-minute interviews, led by experienced qualitative research moderators, were conducted using dedicated discussion guides and a frame-by-frame PDF version of the Pre-CoMiSS™ tool. Thematic analysis of the data was undertaken. **Results**: A total of 20 parents participated, 10 from the UK and 10 from Sweden. The Pre-CoMiSS™ tool was well received by parents from both countries. Parents found the tool’s results clear, useful, and easy to understand. However, some parents, especially from Sweden, expressed the need for additional guidance on next steps. **Conclusions**: Parents found the Pre-CoMiSS™ tool acceptable and easy to use, although some requested clearer guidance on next steps. These findings support further refinement and formal validation before clinical implementation.

## 1. Introduction

Cow’s milk allergy (CMA) is one of the most frequent food allergies in infancy [1], with an overall estimated prevalence of immunoglobulin E (IgE)-mediated CMA ranging from 0.5% to 4.9% in infants [2,3]. This prevalence has been estimated to increase in recent decades, from 2.3% between 2000 and 2012 to 3.9% between 2012 and 2021 [4]. CMA, an immune reaction to cow’s milk proteins, can present with a wide range of gastrointestinal, dermatological and respiratory symptoms [2,5]. Due to the non-specific nature of these symptoms, recognising possible CMA can be challenging for both parents and healthcare professionals (HCPs) [6,7]. Such challenges can pose a significant burden on healthcare systems, risking both under- and overdiagnosis, and can potentially negatively impact wellness and growth in children and quality of life in their caregivers [8,9]. Guidelines recommend that a diagnosis of CMA is confirmed using a diagnostic elimination diet of 2–4 weeks followed by a supervised cow’s milk challenge [10,11,12,13,14]. However, barriers to performing the food challenge include limited clinical resources, the time-consuming nature of the test and parental concern about symptoms returning [6,14].

With the increased use of online resources for health-related issues, the provision of evidence-based online information for parents, including those relating to allergy in young infants, is of paramount importance [15]. Parents have been shown to frequently use general online health information (52% to 98%) and specific online health information relating to their child’s condition (12% to 99%) [16]. Online symptom tracking tools have also become highly accessible; a recent consensus study reported the availability of 10 online questionnaires in English language relating to CMA symptoms [17]. However, most of these tools were non-validated and included different symptoms; experts therefore recommend using only validated tools and involving a HCP in reviewing and interpreting the tools or results [17].

The Cow’s Milk-Related Symptom Score (CoMiSS™) is an awareness tool developed by an expert scientific committee to help HCPs evaluate symptoms potentially related to CMA [18,19]. Since the development of CoMiSS™, the scientific committee has endeavoured to undertake a permanent ‘march’ to accumulate data and reinforce both the validity and usefulness of the tool. The tool assesses gastrointestinal, skin and respiratory symptoms, as well as the occurrence and intensity of regurgitation and crying, with a score of 10 or more out of 33 indicating that assessment for possible CMA might be of benefit to the child [20]. A recent meta-analysis of 14 studies demonstrated that CoMiSS™ has a good level of sensitivity (0.64) and specificity (0.75) and showed a significant improvement in symptoms in these infants following a cow’s milk elimination diet [21]. The CoMiSS™ has been shown to be a predictor for CMA in infants with feeding problems and malnutrition [22].

The role of parents in contributing to the diagnosis of CMA is well recognised by experts [23]. Therefore, the parent-specific CoMiSS™ tool (Pre-CoMiSS™) was developed in 2024 by the CoMiSS™ expert consensus panel, to help parents track and record their baby’s symptoms and to facilitate discussions with their HCP. A qualitative study assessed the need and usefulness of the tool for both parents and primary care physicians [24]. The study found that Pre-CoMiSS™ was well received by both parents and primary care physicians, although some physicians raised concerns regarding parental anxiety and additional workload [24]. While this study looked at the necessity for such a tool, further exploration of the end users’ experience of using and interacting with the tool was needed. Therefore, the objective of this qualitative study was to explore users’ experience with the Pre-CoMiSS™ tool, learn from their experience and identify areas for improvement.

## 2. Materials and Methods

Participants were selected from a local panel of potential respondents interested in participating in market research from across two countries—the UK and Sweden. These two countries were selected based on their familiarity with the concept of CoMiSS™ and their involvement in the first qualitative interviews with parents and HCPs [24]. Ipsos (Geneva, Switzerland; www.ipsos.com (accessed on 27 January 2026)) and its local partners were responsible for generating and continuously refreshing and enriching the local panels via methods such as social media reach or local newspaper announcements. The local panels represent pre-recruited representative individuals from specific geographic areas. The selection of respondents and use of local panels was independent from any of the Pre-CoMiSS™ experts. Potential respondents were recruited by telephone, email or social media, depending on the country. There were no public announcements and paediatricians were not involved in the recruitment phase. All participants went through final screening and scheduling via telephone.

Participants were parents of infants aged up to 12 months who felt that their babies were experiencing symptoms such as crying, regurgitation, changes in stool consistency or skin irritation, and for whom infant formula was introduced in the last 2 months (either mixed breastfeeding and formula or formula only). During the recruitment screening process, parents’ interest in using a symptom-tracking tool was gauged by asking them to select which tool, information page, or search engine they would be interested in using from five options. Once successfully recruited, these parents were instructed to use the Pre-CoMiSS™ tool in a real-world setting in the days leading up to their interview, ensuring they were actual users of the tool at the time of data collection.

Forty-five-minute interviews were conducted by qualitative research moderators with parents from the two countries. UK interviews were conducted in English in June 2025 and formed part of the initial qualitative research. Based on the recommendations from the UK findings, the tool was then updated, and this improved version was subsequently evaluated by parents in Sweden. Interviews in Sweden were conducted in November 2025 in Swedish language. A sim-translator was also used throughout the Swedish interviews, with a human interpreter translating 50% of interviews to English live. Throughout the interviews, visual support was provided in the form of a frame-by-frame PDF version of the Pre-CoMiSS™ tool. The tool and the frame-by-frame portable document were updated prior to the Swedish interviews based on UK learnings; these were both in English and were not translated for the Swedish participants. Interviews were facilitated by dedicated discussion guides. Audio recordings of the interviews were captured and used for the generation of field notes. Field notes were taken during and following the interviews.

Interviews were conducted by two female moderators, one per country, fluent in the local language, who were trained market researchers and experienced qualitative research moderators working in the field for 15 years or more. Participants were informed about the moderator’s first name and that they were working in the field of market research on a project for Nestlé Nutrition and Health (Vevey, Switzerland) via Ipsos. Additionally, participants were informed that the study objective was to gain their feedback on an online tool designed for parents with concerns regarding their baby’s feeding symptoms and reactions. Participants were requested to visit the Pre-CoMiSS™ online tool prior to the interview. Respondents also went through a screening questionnaire containing questions related to CMA and were likely to infer that CMA would be discussed during the interview. A monetary incentive of 60 to 70 euros was provided to participants, regardless of the answers given.

An inductive thematic analysis was conducted, allowing themes to emerge directly from the participants’ narratives rather than relying on a pre-existing framework. The analytical process involved live observation of select interviews by the research team, familiarization with the audio recordings and field notes, generation of initial codes by the local moderators, and a collaborative review to group these codes into overarching themes. No qualitative data analysis software was used; coding and theme generation were conducted manually.

## 3. Results

**Figure 1:**
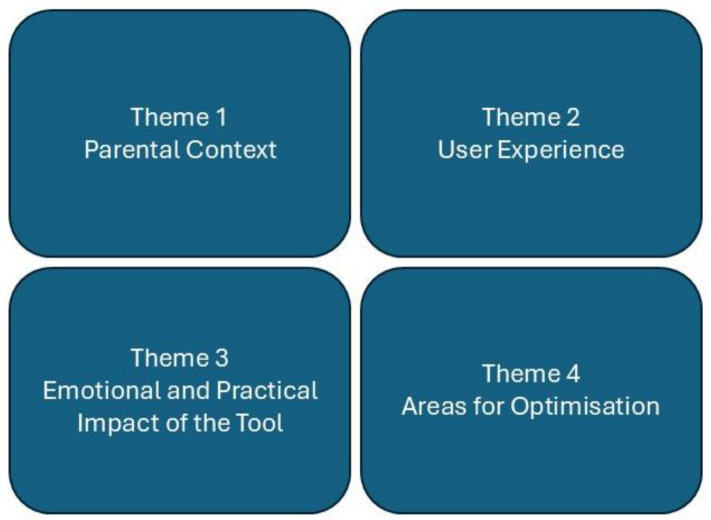
Four themes derived from the data.

A total of 20 interviews were conducted, 10 interviews in the UK and 10 in Sweden. No interviews were deleted or edited; all answers were collected and considered. There were no parents of children with previously confirmed CMA. Details of the participant demographics are shown in Table 1. A total of four themes emerged from the data: parental context, user experience, emotional and practical impact of the tool, and areas for optimisation (Figure 1). Some comments have been selected and reported as quotes to serve as examples of the topic under consideration.

### 3.1. Theme 1: Parental Context

#### 3.1.1. Awareness

There was high awareness of CMA among parents in the UK, often coming from friends or broader social networks. Awareness was lower in Sweden, with parents’ suspicion of milk protein allergy (‘mjölkproteinallergi’) often prompted by a personal or close family history with allergies. In both countries, the term ‘cow’s milk protein allergy’ is rarely used; simpler phrases like ‘reaction to cow’s milk’ in the UK or ‘intolerance’ (‘intolerans’) and ‘milk protein allergy’ (‘mjölkproteinallergi’) in Sweden were used by parents in conversation or when searching online.

“I do actually think that she has CMA. We gave her a bottle of formula at 8 weeks old and she was really sick immediately and refused bottles ever since.”

(Mum, 8-month-old baby, UK)

“I thought that it was a milk protein allergy, isn’t that what it’s called when they are sensitive? But then I was told no, babies can get rashes from anything… I feel like I am investigating this myself.”

(Mum, 6-month-old baby, Sweden)

#### 3.1.2. Concerns

In both countries, parents reported many concerns regarding a potential CMA diagnosis, including dietary inconveniences (the need for specialised formula or a restricted diet), emotional concerns (child missing out on life experiences due to their allergy) and safety risks (such as anaphylactic shock).

“I don’t want her to have an allergy. It will be a bit of a burden from a practical point of view, and I don’t want her to miss out on normal things like enjoying an ice cream with her friends when she gets older.”

(Mum, 10-month-old baby, UK)

#### 3.1.3. Parental Research

In both countries, parents performed their own initial research after noticing symptoms, both online and via conversations with other parents or close family members. Many performed a general Google search (19 out of 20 participants) or explored authority websites such as the UK National Health Service (NHS) website (6 out of 10 participants) or 1177.se (Sweden’s national digital health portal, 10 out of 10 participants) to understand potential causes of symptoms such as rashes and digestive issues. Parenting applications (such as Peanut; www.peanut-app.io/ (accessed on 27 January 2026)), forums (Parenthood ‘Föräldraliv’) or social media (TikTok) were also seen as useful for understanding other people’s experiences, but were recognised as not very trustworthy, especially in Sweden. In Sweden, 2/10 participants used a symptoms list on Knodd, a trusted site for children’s health, while 1/10 in the UK used a non-expert designed and unvalidated symptom checklist that was developed by a nutrition company.

#### 3.1.4. Next Steps

In Sweden, the Child Healthcare Centre ‘Barnavårdscentral’ (BVC) system of clinics was reported as the clear next step after initial research (10 out of 10 participants), while not all of UK participants approached a general practitioner (GP; 8 out of 10 participants). Initially, parents reported undertaking a few adjustments, including a review of their own diet, if breastfeeding, to cut out or reduce dairy or a switch of bottle or teat type. Trial-and-error switching between mainstream formula brands and their ‘sensitive’ variants prior to consulting a HCP was common in Sweden (5 out of 10 participants) but seldom occurred in the UK (2 out of 10 participants). Conversely, independently switching to a specialized CMA formula before HCP consultation was almost non-existent across both countries. Parents approached their appointment with a HCP with a sense of trepidation, often concerned about their baby’s symptoms being the source of something serious and fearing the HCP would dismiss the symptoms as normal. Their experience with their HCP was generally positive when symptoms were taken seriously and further tests or checks were put in place. In the UK, participants used the NHS 24-h helpline, health visitors, pharmacists, lactation consultants, dietitians or the emergency department as resources before going to the HCP. In Sweden, if participants felt unheard by the BVC or in acute cases, the emergency room (Akuten) or online doctor services (Knodd) were used.

“When we’ve been worried, especially out of hours, we’ve called 111. They can reassure you or let you know if it’s serious.”

(Dad, 6-month-old baby, UK)

“You have to pester them […] I think I talked to the BVC every day about the problems. I almost felt nagging and whiny. I almost felt like I was exaggerating.”

(Mum, 6-month-old baby, Sweden)

### 3.2. Theme 2: User Experience

#### 3.2.1. Usability

The tool was seen as clear, easy and quick. Participants reported that the tool guided them through the symptoms without overwhelming them. Breaking the problem down into bite-sized chunks gave parents a sense of calm and progression. Most parents finished using the tool in under 10 min (even less with fewer symptoms), with completion times ranging from 5 to 20 min. The ability to skip non-relevant symptoms was appreciated. Participants reported that the language was simple, with no complex medical terminology; instructions were concise and easy to follow. While English was fine for the Swedish respondents, participants would have preferred a Swedish language version.

“It’s really helpful because the way it’s laid out walks you through each of the different things with babies… It goes through the skin rashes, the poos and other bits that it could be. It’s very useful as well because sometimes you forget, especially when you’re not sleeping.”

(Mum, 6-month-old baby, UK)

“I liked that I didn’t have to type or write anything, just tick boxes or pick from [the] menu.”

(Mum, 10-month-old baby, UK)

“Pictures of symptoms are much appreciated—gives a clear and good understanding for what’s normal.”

(Mum, 5-month-old baby, Sweden)

#### 3.2.2. Relevance

Parents reported that the tool aligned with their expectations, aiding them in symptom tracking and understanding. Most parents were not concerned by the lack of a diagnosis and were eager to use the information gathered using the tool for GP consultations. This gave them a sense of feeling prepared and empowered.

“I can go to the GP with a bit of knowledge behind me now—you have to be quite assertive with them.”

(Mum, 3-month-old baby, UK)

“This would have helped me to remember things when going to the BVC.”

(Mum, 8 m baby, Sweden)

“It’s calming to read what’s normal, the tool adds value.”

(Mum, 7-month-old baby, Sweden)

#### 3.2.3. Design Appeal

Parents reported that the design effectively communicates a medically informed yet approachable tool, using clean, clear colours and reliable, accurate photos that reinforce a professional and medical tone. However, parents suggested the aesthetics of the tool needed improving.

“It looks very neat, it’s not all over the place.”

(Mum, 2-month-old baby, UK)

“The layout of questions, info[rmation] and symptoms looks nice and is easy to understand and follow.”

(Mum, 3-month-old baby, Sweden)

“Visually it didn’t engage me. It looked quite dated and boring—lots of colours does not add professionalism.”

(Mum, 5-month-old baby, Sweden)

#### 3.2.4. Page-by-Page Feedback

Page-by-page feedback is summarised in Table 2.

### 3.3. Theme 3: Emotional and Practical Impact of the Tool

The Pre-CoMiSS™ tool was very well received by parents from both countries. It was seen as highly appealing, distinctive and credible. With no similar tools available, Pre-CoMiSS™ is unique, answers a real and pressing need and felt trustworthy and reliable. Very few participants remarked on the involvement of Nestlé Nutrition and Health but those who did were happy to accept their involvement and acknowledged their depth of expertise in this area. Parents understood and accepted the tool as a symptom recorder/tracker. Most parents were happy to record symptoms and then approach a HCP for a formal diagnosis, if necessary. Parents in both countries appreciated this record of symptoms, feeling prepared and well-equipped for discussions with HCPs. The tool was also found to be useful for multiple audiences, e.g., those who discover that their baby is unlikely to have CMA and those who can see their baby is exhibiting multiple symptoms of CMA.

Moderators asked open-ended questions about how each parents’ emotional state changed after using the Pre-CoMiSS™ tool, specifically probing whether the experience provided reassurance or induced further anxiety. The Pre-CoMiSS™ tool was found to be both empowering and reassuring. After using Pre-CoMiSS™ parents felt calm, reassured, validated, confident, clear-headed, informed, empowered and proactive. Additionally, the tool did not trigger or exacerbate anxiety among the respondents.

“It’s friendly, but informative. It makes me think it’s from a reliable source.”

(Mum, 8-month-old baby, UK)

“I think it equips you better, makes you feel more knowledgeable, like actually you don’t need to freak out so much about this, because it’s normal in babies.”

(Mum, 6-month-old baby, UK)

“They have put my mind at ease. It’s good to have resources that are backed up by medical professionals.”

(Mum, 6-month-old baby, UK)

“It gives me reassurance and something to lean on. Symptoms go back and forth and I can register these and get a quick overview rather than having to wait for the next meeting at BVC.”

(Mum, 9-month-old baby, Sweden)

“This is good information, when googling you more or less read that everyone is going to die for each symptom, sort of. So it’s calming to read that it’s normal that she cries and then get measurement of how much, how long and how old etc.”

(Mum, 5-month-old baby, Sweden)

“I noticed that it’s Nestlé that offer this. They have long experience so I’m not surprised—they know what they are doing.”

(Mum, 5-month-old baby, Sweden)

### 3.4. Theme 4: Areas for Optimisation

Areas for optimisation in each section are described in Table 3.

In both countries, but more prominently in Sweden, some parents asked for guidance on interpretation and a recommendation for the next steps following completion of the tool. In the UK, where parents were often in the early stages of investigating their child’s symptoms and feeling highly concerned, some parents expressed the need for either information on non-CMA symptoms or a stronger mandate to consult their doctor. In Sweden, where parents were often further along and already in dialogue with their BVC, a strong and consistent desire for a more interpretive summary to guide their next action was expressed for 5/10 parents. A couple of parents from each country suggested reframing the output from data record to a guided recommendation. Additionally, some parents expressed that they would like to be able to complete the questionnaire on different dates and compare the summaries side by side. Finally, a further next step is to consider modernising the design of Pre-CoMiSS™ to bolster credibility and user experience by evolving the colour palette and typeface.

## 4. Discussion

The study has provided the opportunity to optimise the Pre-CoMiSS™ tool, based on the experience of parents from two European countries, prior to further refinement and validation. The study forms part of ongoing efforts by the scientific committee to accumulate data and provide evidence for the validity and usefulness of the tool. In-depth qualitative data has been gathered highlighting the importance for parents of having an explanation for their child’s symptoms, exploring parents’ unmet needs and contributing to raising awareness of CMA-associated symptoms among parents.

### 4.1. Parental Context

The Parental Context theme findings show high CMA awareness among UK parents but lower awareness in Swedish parents. Parents used online platforms, such as Google or authority websites, and conversations with other parents or family members as part of their initial research after noticing symptoms. Next steps after initial research were the Child Healthcare Centre, in Sweden, while parents in the UK used other resources before seeing their GP. Other studies have demonstrated poor parental knowledge and awareness of food allergies [25,26]. In a survey of parents of children with a diagnosed food allergy in the Netherlands, a median knowledge score of 9.9 out of 21 was reported; higher parental knowledge of food allergies was associated with higher education level, being a member of a patient organisation, contact or visits with a paediatrician or allergist, and history of anaphylaxis in the child [27]. Improved parental knowledge of food allergies and the removal of barriers to elimination diets are essential for ensuring the effective management of CMA. Confusion surrounding the nomenclature of CMA has also been highlighted in this study. CMA is classified as IgE-mediated and non-IgE mediated types, however, the term “intolerance” is widely used for non-IgE-mediated CMA, by both parents and HCPs. This confusion can delay diagnosis and appropriate management [28,29]. Pre-CoMiSS™ may be able to provide reassurance while raising awareness among parents and encourage discussions with HCPs to ensure accurate diagnosis and symptom management.

Use of online resources by parents for researching healthcare information has been reported in other studies, with up to 90% using Google or similar search engines [15,16]. As such, online questionnaires that raise awareness of CMA have become available in several countries. While these may have benefits, such as the patient being better informed, improved communication between the patient and the physician, and the shared decision-making process, there are concerns about the quality of the resources, the questionnaires causing needless healthcare visits, and affecting the relationship between the patient and HCP [17]. Furthermore, increasing use of artificial intelligence technologies for obtaining information and simplified explanations of medical terminology offers opportunities for patients and caregivers; however, their use comes with risk due to the lack of regulation and oversight [30]. Validated tools and input from an HCP are essential to ensuring that patients access high-quality and reliable information [16]. In terms of parents’ next steps after using the tool, the use of other resources before seeing the GP in the UK may be a result of concerns about wasting doctors’ time, which is a reported barrier to seeking help in the country [31].

### 4.2. User Experience

Findings from the User Experience theme demonstrate that parents were very positive about the Pre-CoMiSS™ tool, with the inclusion of information on what is ‘normal’ being particularly useful and reassuring. The tool was seen as clear, easy and quick, aligning with parent expectations of a tool to track symptoms. The design was reported to effectively communicate a medically informed yet approachable tool, with some need to improve on the aesthetics. Some parents, especially in Sweden, expressed a desire for clearer guidance on what to do next. Evaluation of another parental screening tool relating to child development and well-being has highlighted that online screening tools are seen as positive, especially if they are quick and easy to navigate, and can facilitate open discussion and conversation with HCPs [32]. Similar to our study, parents expected support and guidance following the use of the tool, and understanding what is ‘normal’ was seen as important for parents [32]. Use of the screening tool responses in dynamic discussion with HCPs was also shown to help parents feel secure [32]. However, difficulties in answering questions in a non-native language were found to lead to some level of misunderstanding; this should be taken into consideration when disseminating the Pre-CoMiSS™ tool to non-native English-speaking countries.

Further studies have reported that the use of online resources, in general, help parents prepare for discussions with their HCPs [33]. Parent concerns about what is ‘normal’ were also highlighted as a reason to seek medical advice from HCPs after the use of online resources [34,35]. One study stressed that online resources should be used to complement information from HCPs, highlighting that HCPs can validate parent concerns and help to ease worries associated with their child’s health [34]. Validation of parental concerns about their child’s symptoms has also been found to have a deep impact on parents of infants with regulatory problems [35].

### 4.3. Emotional and Practical Impact of the Tool

Findings from the Emotional and Practical Impact of the Tool theme demonstrated that Pre-CoMiSS™ was seen as a highly appealing and credible tool. With no similar tools available, Pre-CoMiSS™ is unique and answers a real and pressing need. The tool was found to be both empowering and reassuring, helping parents to feel in control and well-equipped for discussions with their HCP. In our previous study, the need for and potential usefulness of the Pre-CoMiSS™ tool was assessed by parents and primary care physicians [24]. The study revealed that Pre-CoMiSS™ was well received by the parents and most physicians; however, there were some concerns about the tool causing anxiety in parents. In this study, we moved from assessing the necessity of the tool towards optimising the tool for end users, a crucial next step towards wider dissemination of Pre-CoMiSS™. Here we report that parents found Pre-CoMiSS™ to be empowering and reassuring and did not trigger or exacerbate anxiety. Nevertheless, the risk of parental over-interpretation or increased anxiety should be further assessed. On the other hand, CMA itself has been associated with high levels of anxiety and depression in caregivers, especially when their children present with respiratory symptoms or anaphylaxis [36]. Food allergy-related anxiety among parents has been shown to affect cognition, behaviour, physical distress, sleep and quality of life [37]. A further study has shown that information on the internet can be a source of anxiety, in 14% to 52% of parents, with many desiring more guidance from the doctor on reliable sources [16].

### 4.4. Areas for Optimisation

Key areas for optimisation highlighted in the Areas for Optimisation theme were modernisation of the tool’s design, increased clarity about the data not being saved and more visible options for downloading/exporting it, and greater guidance on interpretation of the results or a recommendation for the next steps after completion. A clear recommendation based on risk or level of concern was not provided with the tool since parents should always visit their HCP if they are concerned about the health of their infant. The experts stress that the tool allows the collection of important information on infants’ symptoms but that parents should visit their HCP if they have further questions.

### 4.5. Limitations

Despite the strengths of the study, there are several limitations. The study had a small sample size of 20 participants from two countries. Small sample sizes are common to qualitative studies due to the nature and depth of the information gathered and data saturation is likely to occur with a larger sample size. Additionally, the lack of statistical validation, the lack of longitudinal data and the descriptive nature of the results may limit the generalisability of the findings. However, the study aimed to capture in-depth perspectives of Pre-CoMiSS™ users, which can only be done using a qualitative study design. The recruitment of more experienced or motivated participants who were already showing an interest in using Pre-CoMiSS™ via local panels from Ipsos and its partners may have resulted in some level of selection bias. Nevertheless, the panels used by Ipsos are continuously enriched and refreshed to preserve their representativeness. The lack of additional demographic data relating to the participants, such as education levels and socioeconomic status, is a further limitation; these factors may have influenced parental knowledge and perspectives relating to CMA. The inclusion of only two countries in this analysis might limit the generalisability of findings for other countries, especially outside of Europe. Nevertheless, the cultural differences between these two countries have contributed to collecting a variety of opinions. The use of the tool in Swedish parents, who were not native English speakers, may have impacted their responses, despite the parents expressing that they were fine with the tool being in English. An assessment of the Swedish parents’ proficiency in English may have been beneficial in determining the impact on their comprehension of the questions. The study was supported by Nestlé Nutrition and Health, whose medical and scientific committee attended some of the interviews. It cannot be ruled out that this may have influenced parents’ comments on the questionnaire. However, Nestlé Nutrition and Health did not influence the design or conduct of the study, the analysis of the data or interpretation of the results, and their support was seen as positive by parents, who acknowledged their depth of expertise in infant nutrition. It must also be highlighted that a slightly different version of the tool was used in the two countries; the refined version was used only in Sweden. This iterative approach allowed the validation of the effectiveness of the initial improvements while capturing further cross-cultural insights without duplicating feedback on already identified usability issues. Cross-country differences were interpreted where questions remained the same in both versions. Finally, unverified simultaneous translation and the high prevalence of female participants are also potential limitations.

### 4.6. Future Research

Following this positive first user experience with Pre-CoMiSS™, areas for improvement include enhancing the usability and expanding the reach of the Pre-CoMiSS™ tool by optimising the landing page to clarify purpose and guide users and modernising the design to bolster credibility and user experience. Future research could also involve in-depth interviews with HCPs and the comparison of parents’ reporting of symptoms in Pre-CoMiSS™ with HCP evaluation scores in CoMiSS™. Additionally, comparing Pre-CoMiSS™ responses between healthy infants and those with CMA could help to further strengthen the evidence for the usefulness of the tool. Translation into different languages should also be considered, since this is crucial for further expanding Pre-CoMiSS™. Finally, conducting studies in a clinical setting, including the recruitment of patients based on their clinical histories, would be beneficial in further evaluating the Pre-CoMiSS™ tool.

## 5. Conclusions

Parents involved in the study generally found the Pre-CoMiSS™ tool easy to use and helpful, although some wanted clearer guidance on next steps. These findings suggest that the Pre-CoMiSS™ tool may support parents in recording symptoms potentially related to CMA and facilitate discussions with HCPs.

## Figures and Tables

**Table 1:** Participant demographics.

#	Country	Gender	Age (Years)	Number of Children	Child’s Age (Months)	Working Status	Household Income ^1^	Current Feeding	Observed Symptoms		
									Digestive ^2^	Regurgitation	Other
1	UK	Female	27	1	2	Maternity leave	5–7	IF	Yes	No	-
2	UK	Female	32	1	2	Maternity leave	5–7	IF	Yes	No	-
3	UK	Female	40	2	6	Maternity leave	7–10	BM + IF	Yes	Yes	-
4	UK	Female	36	2	5	Maternity leave	5–7	BM + IF	Yes	No	-
5	UK	Female	34	2	7	Maternity leave	5–7	IF	Yes	No	-
6	UK	Female	34	1	10	Maternity leave	5–7	BM + IF	Yes	Yes	-
7	UK	Female	34	2	8	Maternity leave	10+	IF	Yes	Yes	-
8	UK	Female	36	1	3	Maternity leave	5–7	IF	Yes	No	-
9	UK	Female	35	2	8	Maternity leave	5–7	BM + IF	Yes	No	-
10	UK	Male	31	1	4	Full time	3–5	IF	Yes	No	-
11	Sweden	Female	29	1	9.5	Parental leave	>100	IF	No	No	Yes ^3^
12	Sweden	Female	41	1	3.5	Parental leave	>75	BM + IF	Yes	Yes	Yes ^4^
13	Sweden	Female	30	3	7	Parental leave	>75	IF	Yes	No	-
14	Sweden	Female	40	2	11	Parental leave	>75	IF	Yes	No	-
15	Sweden	Female	43	1	7	Parental leave	>75	IF	Yes	No	-
16	Sweden	Female	33	2	8	Parental leave	>75	IF	Yes	No	-
17	Sweden	Female	31	1	5	Parental leave	>75	BM + IF	Yes	Yes	-
18	Sweden	Female	27	1	2	Parental leave	>75	IF	Yes	No	-
19	Sweden	Female	32	1	2	Parental leave	>75	IF	Yes	No	-
20	Sweden	Female	40	2	6	Parental leave	<75	IF	Yes	Yes	-

^1^ Currency is British Pounds (thousands) per month for UK participants and Swedish Krona (thousands) per month for Swedish participants; ^2^ Diarrhoea, vomiting, constipation or reflux; ^3^ Rashes; ^4^ Noisy breathing, cough, runny nose; BM, breast milk; IF, infant formula.

**Table 2:** Summary of the page-by-page feedback.

Overall Comments	UK	Sweden
Landing page **Works well; is simple and informative.** Clear explanation of role of the tool: ‘to help you understand symptoms’ Reassuringly credible: ‘expert designed’ Quick summary of key symptoms helps parents know what to expect from questions and to feel reassured about their relevance	No additional comments	Does not clearly state what to do: ‘record your baby’s symptoms using Pre-CoMiSS™’ is overlooked or not clear. Some didn’t understand the tool was a quiz/questionnaire. The colour hierarchy creates a usability issue, making the ‘Start’ button difficult to find.
	“It looks very professional. They’ve obviously thought about it carefully” (Dad, 6-month-old baby, UK)	“Intro[duction] with info[rmation] about [the tool being] a test/quiz is needed. If I had not participated in the research, I would not have known what to do and why” (Mum, 9-month-old baby, Sweden) “Your baby’s information will not be saved… should be on the start ‘page’, I want to know this at the start” (Mum, 7-month-old baby, Sweden)
Pre-check **Questions are clear and relevant.** All 7 questions make sense and are seen as important in establishing context Quick and straightforward to complete, no delay in getting to the key questions regarding symptoms	‘Pre-check’ questions feel alienating, as if parents are going to be excluded from the questionnaire if they do not answer correctly 1 or 2 respondents had difficulty selecting the right options from drop-down menus when accessing via a mobile phone (impacting the accuracy of the summary they receive at the end)	No additional comments
	“The questions made sense. I resonated with everything that was there” (Mum, 7-month-old baby, UK)	“Good and clear background questions. It’s easy to understand why these are here” (Mum, 7-month-old baby, Sweden)
Crying **Questions are relatively easy to complete.** The ‘What is normal?’ information is both useful and reassuring Parents found it difficult to accurately calculate total time crying over 1 day	Questions could be made more relevant and reflective of the problem, to help parents feel they are providing more precise answers that better capture the nature and feeding-related timing of crying Slider device is easy to use Some were reluctant to record total crying since not all crying is related to feeding and may not be relevant One Mum realises here that her baby does not cry and therefore her issues are related to her skin and not to feeding; this gives her immediate reassurance (eliminates CMA as an issue in her mind)	Scale works well and gives a good overview ‘Important before completing’ is also valuable information and reassuring, aligned with information from 1177.se and BVC
	“I found it quite hard to add up how long she spends crying over a whole day” (Mum, 10-month-old baby, UK)	“This scale is very good! You don’t really know how much they (babies) cry, and it’s hard to measure, so this is very helpful” (Mum, 5-month-old baby, Sweden)
Spitting up (UK)/Regurgitation (SWEDEN) **Measures provide helpful guidance.** Guidance on volume (teaspoon, half the feed, complete feed) is easy to understand as a measure	Seen as an important question, but not always easy to answer and some wished for more nuanced response options Measures are not consistent (from per day to per feed) Some prefer to refer to this as ‘being sick/vomiting/regurgitating’, which they differentiate from spitting up (a more normal occurrence and less of a cause for concern)	The scale offers a good indication and frame of reference since spitting up often appears more in volume; this is one of the most prevalent symptoms The number of episodes is also an indication that gives good guidance Some slight wording confusion: while all understood what the ‘regurgitating’ section was asking, Swedish mums used the word ‘vomiting’ (kräkningar) in their speech to convey a larger amount (while ‘spitting up’ being lesser in amount than regurgitation)
	“I wasn’t sure if I was meant to answer per day or per feed or if it was asking about her being properly sick (from her stomach) or just spitting back out (from her mouth)” (Mum, 7-month-old, UK)	“The scale is good! 5 mL does not seem much, but it looks like it’s more” (Mum, 3-month-old, Sweden) “I liked the measurement but also how many episodes per day. If it’s 0–2 per day and if it’s after each feeding, I think this was good too” (Mum, 8-month-old, Sweden)
Poo **Photos are highly useful and reassuring, but some parents still struggled to choose the right one.** Often spontaneously recalled as the most impactful and helpful question thanks to clear photos Most find this easy to answer, thanks to specific photos, especially when multiple choices are available	One respondent was relieved to recognise her baby’s ‘green poo’ in the photos as she struggled to describe this to HCPs Some expressed frustration/confusion that their baby’s poos vary so much day by day (especially those who recently started weaning) and that multiple answers would apply so they do not know which to pick as most representative ‘What’s normal’ does not give details on what to expect for infant formula-fed babies (only breastfed), failing to allay the concerns for formula-fed babies	Much appreciated gallery of poo that visualises a good range of what is encountered Useful when describing this to HCPs A few would like to add one or two pictures between picture 3 and 4 and between 4 and 5—the contrast is perceived as too big between the 2 options, hence difficult for some parents to choose the ‘right one’
	“It was really useful to have the pictures; it made it so much easier” (Mum, 8-month-old baby, UK) “It really helped to recognise her poos and to realise that you’re not the only parent experiencing this” (Dad, 6-month-old baby, UK)	“It was very useful and informative to see pictures; it made it so much easier to get an understanding when having a frame of reference. The only thing was that my son was between two pictures and then I did not know which one to choose” (Mum, 8-month-old baby, Sweden)
Skin	**Questions work well, but some struggled to define dry vs. rough vs. itchy.** Easy to choose which area of body is affected and if skin issues affect sleep, but difficult to distinguish between dry, rough and itchy No photos or information available to show how rashes present on ethnic skin—this alienates some parents	**Visualisation is appreciated but ability to enlarge pictures would be useful.** Most found it easy to understand that different types of skin issues can be part of the symptoms The pictures made it easy to understand the difference between different skin issues; the most illustrative pictures were rash and itchy Some had difficulty distinguishing between dry and rough skin
	“I ticked ‘yes’ because her rash is dry and rough, but it isn’t itchy” (Mum, 6-month-old baby, UK) “I’ve got dark skin. When you have dark skin, rashes sometimes look different. So that probably needs to be noted” (Mum, 10-month-old baby, UK)	
Respiratory **Respiratory symptoms section is unexpected, but valuable learning moment for parents.** Questions were clear and straightforward to answer Some were pleasantly surprised to be asked about respiratory symptoms; they did not necessarily link these with potential food allergies It was good to be reassured that some respiratory issues are normal in babies, and there is no need for concern	No additional comments	No additional comments
	“It helps a lot that it says it’s a common problem in babies. If that wasn’t there, then maybe I would have panicked a little bit” (Mum, 2-month-old baby, UK)	“I thought that it could only affect the breathing, not that it could be a cough or a runny nose. So I thought that was good, I learned something new” (Mum, 8-month-old baby, Sweden)
Results (UK)/Summary (SWEDEN) **Page works well, but some parents still unsure what to do next.** A concise record of information all in one place Not all parents noticed the option to download/export results or failed to read that their data would not be saved Some wanted more overt direction, e.g., if answers indicate a risk of CMA, they would like to be advised to go to their HCP 1/10 in each country expressed a desire to get a diagnostic as an output	No risk of forgetting when nervous or exhausted in front of GP Only one (very calm and non-anxious/sleep-deprived) mum rejected the summary as she had no problems remembering the symptoms herself. Instead, she wanted more direction on what to do next (information and resources or recommendation to visit GP)	Several parents completed the tool a number of times to capture change in symptom patterns over a few days
	“It’s helpful to see it summarised like that, for sure” (Mum, 6-month-old baby, UK)	(Taking the results to HCP) “Yes, I might have had a different dialogue at the BVC, and then maybe we would have set up some other tests or gotten some other info” (Mum, 9-month-old, Sweden)

BVC, ‘Barnavårdscentral’ [Swedish Child Healthcare Centre]; CMA, cow’s milk allergy; GP, general practitioner; HCP, healthcare professional; Pre-CoMiSS, Parent-Specific Cow’s Milk-Related Symptom Score; UK, United Kingdom.

**Table 3:** Summary of the areas for optimisation.

Section	UK	Sweden
Landing page	Opportunity to have more details visible on screen, rather than requiring 4 separate clicks to expand each section (‘Purpose’, ‘How to use’, ‘Symptoms’ and ‘Important before beginning to complete’) Suggest that ‘Important before completing’ details be fully visible and not hidden in a clickable box ☑	Rework the page to explicitly frame the tool as an interactive ‘symptom quiz’ or ‘test’ Enhance the visual hierarchy to make the ‘start’ button a clear, unmissable focal point Move critical information about data not being saved to the landing page to set user expectations upfront Place ‘important before completing’ above the start button to create a more concentrated ‘starting information’ space and increase visibility Include disclaimer about personal information not being saved (currently only included in the summary)
Pre-check	Use more inviting, inclusive and caring language to describe the purpose of the ‘pre-check’ questions, e.g., ‘Tell us about your baby’ ☑ Mobile phone interface: improve to make drop-down menus easier to use ☑	No optimisations suggested
Crying	Timing of crying related to feeding would be a more relevant indication of a problem	‘Important before completing’ should not be repeated as a pop up and on the crying page; this repetition creates too much text
Spitting up (UK)/ Regurgitation (Sweden)	A more consistent scale to be used; asking separately about frequency and then about volume is recommended A slider could be used	No optimisations suggested
Poo	No optimisations suggested	No optimisations suggested
Skin	Include photos of different types of rashes, especially of dry, itchy or rough rashes, to help parents better identify and distinguish between them. Also include ‘hives’/swelling (urticaria) ☑ Include photos or information on how rashes present on ethnic skin ☑	An ‘enlarge’ feature might be useful to help parents better identify and distinguish between skin issues, especially between dry skin and rough skin
Respiratory	No optimisations suggested	No optimisations suggested
Results (UK)/ Summary (Sweden)	More visible option to download/export results so information is not lost ☑ More overt direction on what to do next, e.g., if answers indicate a likely risk of CMA, they would like to be advised to go to their HCP	Make it clearer that data will not be saved and parents should download/export results More overt direction on what to do next, e.g., if answers indicate a likely risk of CMA, they would like to be advised to go to their HCP

☑ Implemented following UK interviews; ☑ Partially implemented following UK interviews; CMA, cow’s milk allergy; HCP, healthcare professional.

## Data Availability

The original contributions presented in this study are included in the article. Further inquiries can be directed to the corresponding author.

## Abbreviations

The following abbreviations are used in this manuscript:

BM	Breast milk
BVC	Barnavårdscentral (Sewdish Child Healthcare Centre)
CMA	Cow’s Milk Allergy
CoMiSS	Cow’s Milk-related Symptom Score
GP	General practitioner
HCP	Healthcare professional
IF	Infant formula
IgE	Immunoglobulin E
NHS	National Health Service
Pre-CoMiSS	Parent-reported Cow’s Milk-related Symptom Score
UK	United Kingdom
